# Probiotic supplementation preserves oxygen saturation during severe hypobaric hypoxia in lowland and high-altitude populations

**DOI:** 10.1016/j.isci.2026.117305

**Published:** 2026-08-27

**Authors:** Vittore Verratti, Massimiliano Marazzato, Pierpaolo Prosperi, Kiara Fiorella Abad Bruzzo, Gianpietro Verza, Simona Mrakic-Sposta, Manuel Marzola

**Affiliations:** 1 Department of Sciences, University “G. D’Annunzio” of Chieti-Pescara, Center for Disability, Rehabilitation and Sports Medicine (CARES), 66100 Chieti, Italy; 2 Italian Society of Mountain Medicine, SIMeM, Padua, Italy; 3 Department of Public Health and Infectious Diseases, Sapienza University of Rome, 00185 Rome, Italy; 4 Department of Pneumology and Respiratory Physiopathology, S. Spirito Hospital, 65122 Pescara, Italy; 5 Independent Researcher, Corso Umberto I 219, Casoli 66043 Chieti, Italy; 6 National Research Council (CNR), Piazzale Aldo Moro,7, Rome, Italy; 7 Institute of Clinical Physiology, National Research Council (IFC-CNR), Piazza Dell’Ospedale Maggiore, 3, 20162 Milan, Italy; 8 Department of Medicine and Aging Sciences, University “G. D’Annunzio” Chieti-Pescara, 66100 Chieti, Italy

**Keywords:** hypobaric hypoxia, high altitude, SpO_2_, AMS, probiotics, acclimatization

## Abstract

Hypobaric hypoxia (HH) reduces SpO_2_, impairing physiological acclimatization responses and performance. The multi-strain probiotic Oxxyslab has been hypothesized to support altitude acclimatization by optimizing systemic oxygen availability. This is theorized to occur through the modulation of the HIF-1α pathway, which may reduce localized intestinal oxygen consumption, potentially mitigating tissue hypoxia. A randomized, double-blind trial at 5,050 m evaluated the probiotic effects on nocturnal SpO_2_ and acute mountain sickness (AMS) incidence. Twenty-one Caucasian lowland residents and eight Nepalese high-altitude porters were enrolled and stratified by treatment. Participants underwent continuous nocturnal SpO_2_ monitoring over different altitudes. The probiotic intervention attenuated hypoxemia at peak altitude, yielding SpO_2_ increases of 5.6% in Caucasian and 4.3% in Nepalese porters of Rai and Tamang ethnicity compared with placebo. AMS incidence remained comparable between groups. These findings show that this multi-strain formulation can ameliorate high-altitude hypoxemia in both investigated cohorts, supporting the proof of concept for non-pharmacological adjuncts to acclimatization protocols.

## Highlights

• Probiotic Oxxyslab attenuated hypoxemia at 5,050 m in both cohorts
• Treated Italians had 5.6% higher SpO_2_ at peak altitude vs. placebo
• Nepalese porters showed 4.3% higher SpO_2_ than placebo
• AMS incidence remained comparable between treated and placebo groups

## Graphical abstract

**Figure:**
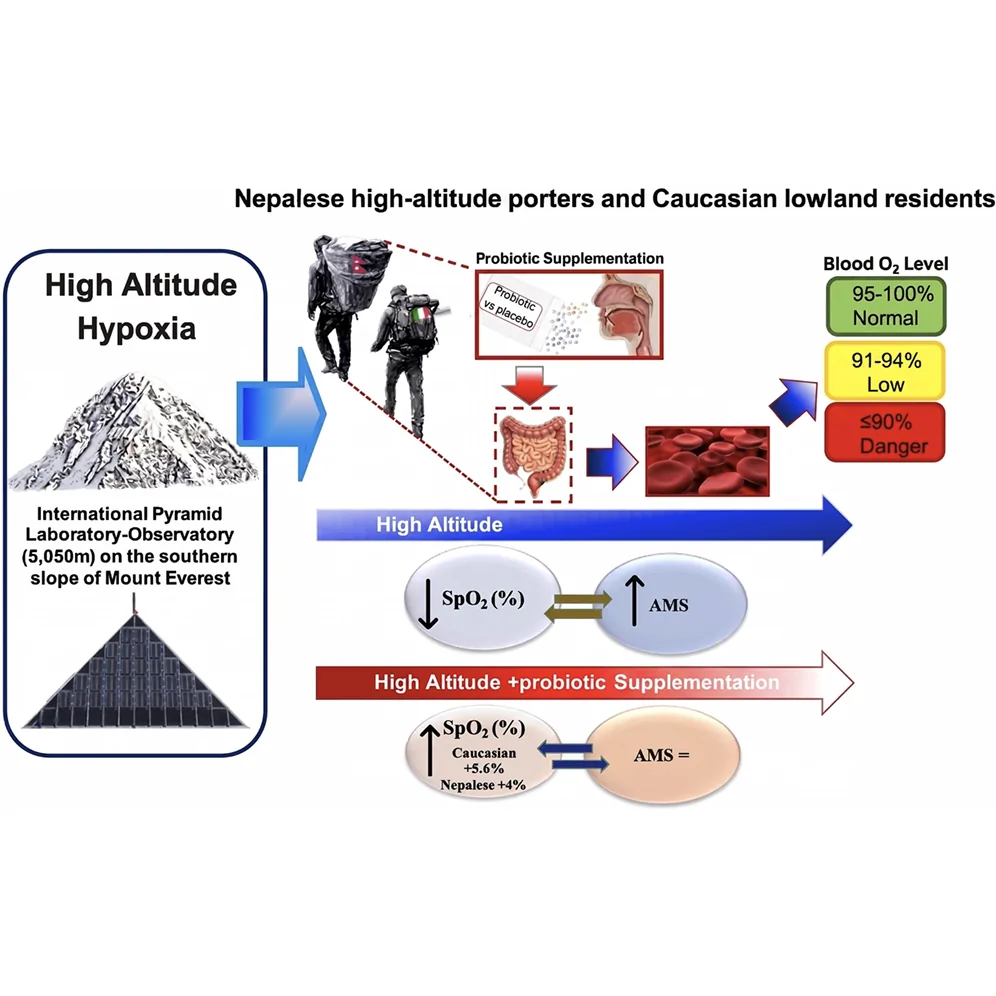

## Teaser

Health sciences; Medicine; Internal medicine; Medical substance; Dietary supplement

## Introduction

Roughly 81.6 million people live at elevations of 2,500 m or higher, predominantly outside Western countries.^1^ As of 2013, it was estimated that tens of millions more people travel to high-altitude regions each year for work or recreation.^2^ More precisely, in some reports, over 100 million lowlanders visit mountainous areas above 2,500 m annually.^3^

Exposure to high-altitude environments causes hypobaric hypoxia, which has a significant impact on human physiology.^4^ Whereas high-altitude natives are chronically exposed to altitude and its associated environmental conditions, high-altitude travelers (e.g., trekkers and climbers) lack prior acclimatization, resulting in exposure to high altitude and hypoxia.^5^ Furthermore, it is important to distinguish between acclimatization, the reversible physiological adjustments made by lowlanders ascending to high altitude, and genetic adaptation, the permanent evolutionary traits that allow certain populations to live permanently at altitudes of up to 5,000 m.^3^^,^^6^

As altitude increases, the partial pressure of oxygen falls, resulting in lower arterial oxygen saturation (SpO_2_) and lower oxygen (O_2_) delivery to tissues, and hypoxic stress triggers a variety of physiological responses aimed at maintaining sufficient oxygen delivery to tissues.^2^^,^^7^ These responses are primarily mediated by the hypoxia-inducible factor (HIF) pathway, a key mediator of cellular responses to low O_2_ availability, capable of regulating various physiological countermeasures, including angiogenesis, metabolic adaptation, and cellular survival.^8^^,^^9^

When these physiological compensatory mechanisms are insufficient to fully restore oxygen balance, negative clinical outcomes emerge; as a result, fatigue and reduced exercise capacity are common with ascent.^10^^,^^11^^,^^12^ With increasing altitude, individuals may develop acute mountain sickness (AMS), and the incidence can vary according to the rate of ascent, altitude reached, and individual susceptibility.^13^^,^^14^^,^^15^ Notably, it is estimated that approximately 25% of individuals experience AMS at altitudes around 3,500 m^16^; in extreme cases, high-altitude pulmonary edema (HAPE) or high-altitude cerebral edema (HACE) can also occur.^13^^,^^14^ A crucial factor in the development of these physiological and pathological conditions is the reduced uptake and subsequent distribution of oxygen at the tissue and cellular levels, which is directly related to the oxygen respiratory gradient. The risk of developing high-altitude illnesses is assessed through continuous monitoring of oxyhemoglobin saturation, which is a useful indicator of both acclimatization and performance at high altitude.^17^

Despite growing interest in developing novel strategies and ergogenic aids to facilitate high-altitude acclimatization and mitigate altitude-related pathophysiology, the therapeutic landscape remains markedly limited. While various gas mixtures and nutritional supplements have shown preliminary promise in experimental settings, the current evidence base for effective interventions is notably sparse. Presently, only two approaches possess substantial clinical validation: gradual, staged ascent protocols and prophylactic acetazolamide administration. However, both interventions present considerable limitations that constrain their practical utility and effectiveness. For instance, although staged ascent protocols are generally protective when feasible, individual variability in acclimatization capacity remains poorly predictable; in fact, some individuals develop altitude illness despite adherence to recommended ascent rates, and the optimal rate has never been definitively established in controlled trials.^18^^,^^19^^,^^20^

On the other hand, although acetazolamide has demonstrated effectiveness in reducing AMS incidence, it is associated with a substantial adverse effect profile that limits its widespread use. The medication induces paresthesias in approximately 50% of users, alongside frequent complaints of dysgeusia, polyuria, fatigue, and gastrointestinal disturbances.^21^^,^^22^ More critically, acetazolamide may carry multiple absolute contraindications, including cirrhosis, severe renal or hepatic dysfunction, electrolyte imbalances, and sulfonamide hypersensitivity, which preclude its use in significant patient populations^23^^,^^24^^,^^25^; rare but severe complications include aplastic anemia, Stevens-Johnson syndrome, and nephrolithiasis, reported in approximately 1%–2% of patients.^23^^,^^24^

In stark contrast to these established but imperfect interventions, widely promoted alternatives, including pre-acclimatization in normobaric hypoxic chambers, specialized gas mixtures, and the majority of commercially available nutritional supplements, lack rigorous scientific substantiation.^14^^,^^26^^,^^27^^,^^28^ This confluence of practical limitations and the absence of validated alternative therapies represents a critical gap in high-altitude medicine, particularly for populations requiring reliable enhancement of physical performance or accelerated acclimatization in hypobaric hypoxic environments.

Recent evidence suggests that a specific probiotic intervention may enhance human adaptation to hypoxic conditions across diverse physiological contexts.^29^^,^^30^^,^^31^^,^^32^^,^^33^^,^^34^ Oxxyslab (formerly SLAB51), an eight-strain probiotic formulation composed of lactic acid bacteria and bifidobacteria, has demonstrated the capacity to induce an oxygen-sparing metabolic response in the intestinal compartment. This formulation promotes adaptive mechanisms in intestinal epithelial cells, enabling enhanced tolerance to hypoxic microenvironments and consequently reducing local oxygen consumption. The reduction in intestinal oxygen demand results in improved systemic oxygen bioavailability, potentially redirecting this critical resource toward organs with higher metabolic priority during acute stress responses, notably the brain, myocardium, and skeletal muscle. This metabolic redistribution may prove particularly relevant for sustaining cognitive function, cardiac performance, and physical capacity under hypoxic challenge.

Oxxyslab has demonstrated improvements in systemic oxygenation across various pathological conditions characterized by hypoxic environments,^29^^,^^30^^,^^31^^,^^32^^,^^35^ as well as in healthy individuals exposed to hypobaric hypoxia. In a randomized, double-blind trial at 3,800 m (White Mountain), participants receiving the probiotic had higher daytime SpO_2_ and nighttime SpO_2_, as well as lower AMS scores than those receiving placebo, with exploratory evidence of augmented ventilatory responses during hypoxia and hypoxic hypercapnic challenge that were associated with a reduced incidence of AMS.^33^

To further confirm and strengthen the evidence for the oxygen-enhancing effects of probiotic Oxxyslab in high-altitude environments, this randomized double-blinded study aims to evaluate the impact of Oxxyslab supplementation on blood oxygen saturation and the occurrence of AMS during a scientific expedition conducted under severe hypoxic conditions across individuals of different ethnic populations (Italian trekkers and Nepali cohort).

## Results

**Figure 1:**
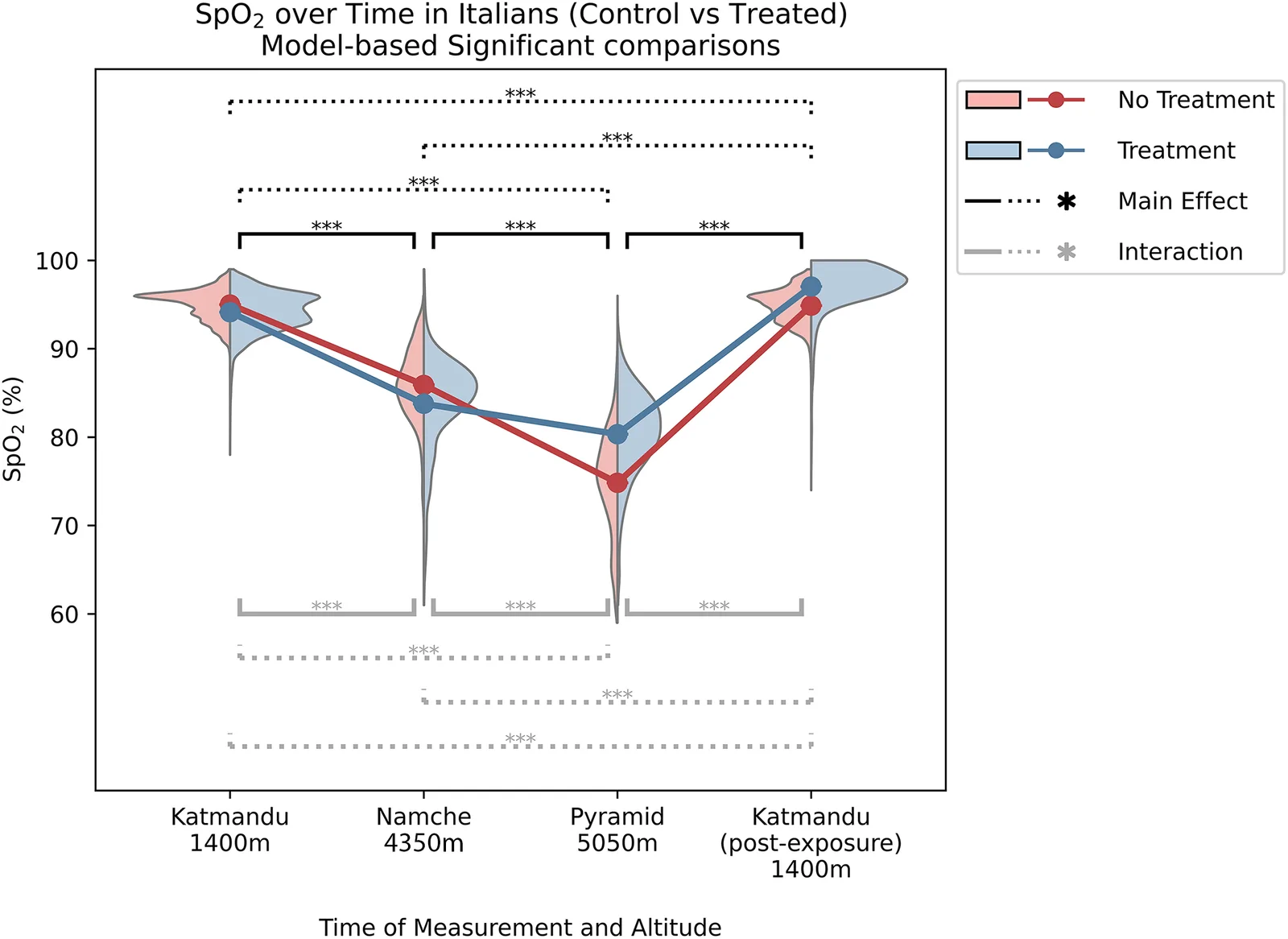
Nocturnal blood oxygen saturation over time in the Italian cohort (control vs. treatment) Model-based significant comparisons of continuous SpO_2_ (%) across four altimetric time points: baseline in Kathmandu (I-T0), mid-altitude in Namche (I-T1, 3,450 m), peak altitude at the Pyramid Laboratory (I-T2, 5,050 m), and upon return to Kathmandu (I-T3). The probiotic-treated group demonstrated a significantly attenuated decline in SpO_2_ during severe hypoxia, maintaining a 5.6 percentage-point advantage over the placebo control group at peak altitude. Furthermore, the treated group showed a more robust recovery post-trek (I-T3), exceeding their own baseline levels. ∗∗∗ = *p* < 0.001.

Blood oxygen saturation (SpO_2_) among Italian participants varied significantly over time (*p* < 0.001), with differences observed between all measurement points, including between baseline (Kathmandu pre, I-T0) and after trek (Kathmandu post, I-T3) (*p* < 0.001). These changes were consistent across both treatment and placebo groups (Figure 1).

The SpO_2_ values over time showed significant differences between the treated and placebo (control) groups, as indicated by a statistically significant interaction between treatment and time (*p* < 0.001). In particular, the treated group exhibited a more attenuated decline and a more robust recovery compared to the control group. A direct comparison of the mean SpO_2_ values highlights the evolving effect of the treatment. Although the control group’s SpO_2_ was initially higher at Namche (I-T1) by 2.6 percentage points (85.8% ± 2.6% vs. 83.2% ± 3%), this dynamic reversed at peak altitude (I-T2). At this point of greatest desaturation, the treated group maintained a SpO_2_ that was 5.6 percentage points higher than that of the control group (80.3% ± 3.1% vs. 74.7% ± 3.2%). This benefit was maintained during the recovery phase (I-T3), when the treated group’s SpO_2_ increased above its own baseline levels and outperformed the control group by 2.3 percentage points (97.2% ± 1.1% vs. 94.9% ± 1.3%).

**Figure 2:**
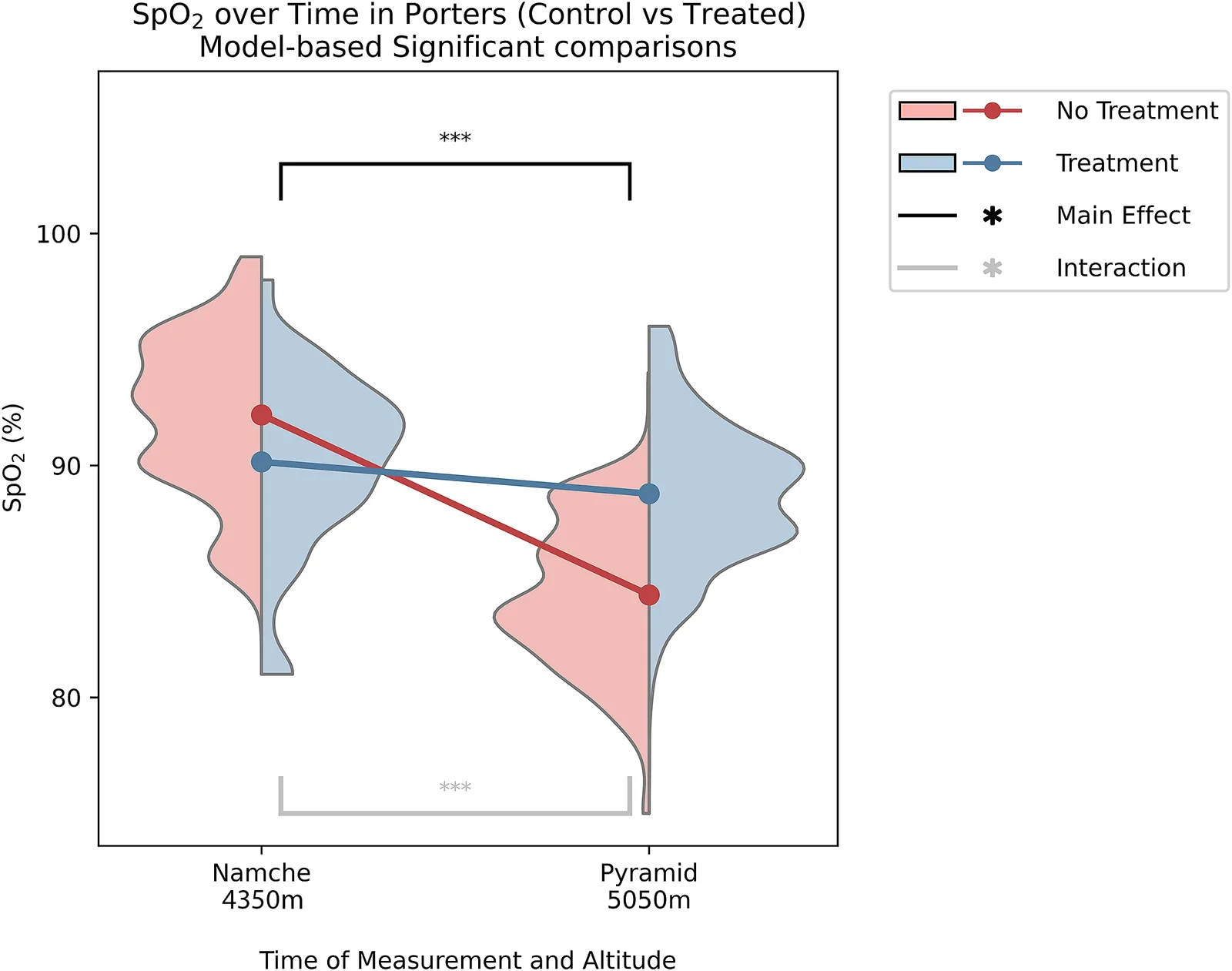
Nocturnal blood oxygen saturation over time in the Nepalese porter cohort (control vs. treatment) Model-based significant comparisons of SpO_2_ (%) across habitual altitude of residence (N-T0) and the peak altitude (N-T2). At severe hypoxia (N-T2), the probiotic treatment significantly attenuated the oxygen saturation decline, yielding a 4.3 percentage-point advantage compared to the control group among the Rai and Tamang porters. ∗∗∗ = *p* < 0.001.

In the Nepalese porters (Rai and Tamang), probiotic treatment significantly attenuated the decline in oxygen saturation in severe hypoxia (peak altitude, N-T2) compared with the control, with an average difference of 4.3% (*p* < 0.001). In particular, while the untreated group experienced a drop of 7.7 percentage points (from 92.2% ± 2.9% to 84.5% ± 2.8%), the treated group showed a minimal reduction of 1.3 percentage points (from 90.1% ± 3.3% to 88.8% ± 2.9%) (Figure 2).

Leave-one-out (LOO) sensitivity analyses confirmed the robustness of the primary findings against individual participant influence. In the Italian cohort, the treatment effect at peak altitude (T2) remained remarkably stable and statistically significant across all 21 iterations, with coefficients ranging from 5.42 to 7.23 (all *p* < 0.05) (Figure S1). The Nepalese porter cohort exhibited similar stability across all 8 iterations, with the T2 interaction coefficient ranging from 4.97 to 7.49 (all *p* < 0.001) (Figure S2). These analyses demonstrate that the observed physiological benefits are robust and not driven by individual outliers despite the limited sample size. These analyses confirm that the previously discussed absolute SpO_2_ advantages at 5,050 m, amounting to ∼5.6% in the Italian cohort and ∼4.3% in the Nepalese porters (Rai and Tamang), are robust population-level effects and not artifacts driven by individual outliers.

The Lack Louise test showed positivity rates of 14.3%, 33.3%, and 14.3% at Namche (3,450 m), at Dingboche (4,320 m), and at Pyramid International Laboratory (5,050 m), respectively. Tests are summarized in Table 1. In the Italian cohort, a generalized linear mixed-effects model was applied to assess the probability of testing positive over time and across treatment conditions. No statistically significant differences were found between the two groups (*p* = 0.729), across time points (*p* = 0.287), or in the interaction between group and time (*p* = 0.409).

**Table 1:** Lack Louise positivity test over three times

Condition	Namche (3,450 m)	Dingboche (4,320 m)	Pyramid laboratory (5,050 m)
C	/	/	/
T	P (3 points)	P (3 points)	/
C	/	P (4 points)	/
C	/	/	/
T	/	P (3 points)	/
T	/	/	/
T	/	/	/
C	/	/	/
C	/	/	P (3 points)
T	/	/	/
T	/	P (3 points)	/
C	/	/	/
C	/	/	P (3 points)
T	/	/	/
T	/	P (3 points)	/
T	P (3 points)	P (5 points)	P (8 points)
C	/	/	/
C	P (3 points)	P (4 points)	/
T	/	/	/
C	/	/	/
T	/	/	/
SUM T	2P (6 points)	5P (17 points)	1P (8 points)
SUM C	1P (3 points)	1P (4 points)	2P (6 points)

C, control group; T, treated group; light positivity P (3–5 points), mild positivity P (6–9 points), severe positivity P (≥10 points), and /, negativity.

At 3,450 and 4,320 m, treated participants exhibited slightly higher positivity rates than the placebo group, although these differences were not statistically significant. At 5,050 m, this trend reversed, with the placebo group showing marginally higher positivity than the treated group. However, no time point showed a significant difference between groups.

## Discussion

### Main findings

The present study was designed to evaluate the effects of Oxxyslab during prolonged exposure to hypobaric hypoxia, combined with physical exertion (trekking), in both subjects unaccustomed to living at high altitude and individuals accustomed to being exposed to such conditions. The objective was to explore not only the effectiveness of a specific probiotic formulation to improve acclimatization at high altitude but also to evaluate potential differences in efficacy related to physiological variability. The present findings expand current altitude acclimatization models, suggesting that interventions may involve alternative pathways, such as a gut-systemic oxygen redistribution pathway. The demonstration that a gut-targeted probiotic intervention can substantially preserve arterial oxygen saturation during severe hypobaric hypoxia, yielding a 5.6 percentage-point advantage in lowlanders and a 4.3 percentage-point advantage in the Nepalese cohort at 5,050 m, represents a relevant finding. In fact, while SpO_2_ differences of ≤3% are generally attributable to measurement artifacts, a modest reduction in peripheral saturation is widely associated with higher AMS risk, cognitive and physical impairment, and reduced exercise capacity.^36^^,^^37^^,^^38^

### Proposed mechanisms of action

The magnitude of effect observed here, particularly the preservation of SpO_2_ above 80% in the treatment group compared to with 74.7% in controls at peak altitude, is comparable to improvements reported with some established pharmacological interventions. This 5.6% differential in Italian participants may represent a clinically meaningful threshold that helps maintain functional capacity at extreme altitudes. These observations warrant careful consideration as they suggest potential alternative pathways for oxygen allocation during hypoxic stress that complement existing physiological models.

Existing physiological models demonstrate that splanchnic oxygen extraction and hemodynamics can be modulated during systemic stress,^39^^,^^40^ and recent *in vitro* observations have established that Oxxyslab induces HIF-1α stabilization via dual inhibition of PHD2 and activation of PI3K/AKT signaling in intestinal epithelial cells under normoxic conditions.^29^^,^^41^

We hypothesize that the splanchnic circulation may act as an active regulatory node capable of strategic oxygen conservation. Although our clinical study design does not directly measure splanchnic hemodynamics or intestinal metabolism, we theorize that this formulation may promote a metabolic preconditioning state. By reducing local intestinal oxygen consumption through enhanced glycolytic flux, this process could theoretically increase systemic oxygen bioavailability, redirecting it to organs with higher metabolic priority during a hypoxic challenge. Future experimental studies are required to directly validate this proposed mechanism.

### Population differences

The different response between the Italian and Nepalese cohorts (5.6% vs. 4.3%) merits a cautious interpretation. While intrinsic differences in the study design preclude a formal statistical comparison and limit us to an exploratory observation, the results suggest a consistent physiological response to severe high-altitude exposure across both groups. The data indicate that the probiotic intervention can provide measurable oxygen-sparing benefits not only to unacclimatized lowlanders but also to individuals who are chronically exposed to high-altitude environments.^42^

### Clinical and translational implications

Whereas pharmacological prophylaxis and acclimatization protocols exhibit substantial inter-individual variability in efficacy, the present data suggest that gut-mediated oxygen sparing may represent a mechanism to support these robust strategies and enhance their effectiveness. In addition, the most intriguing observation concerns the persistence of elevated SpO_2_ in the treatment group upon return to baseline altitude (I-T3), where treated participants maintained levels 3.3 percentage points above baseline while controls returned to pre-expedition values. This sustained enhancement, observed 8 days after the final probiotic administration, cannot be readily explained by acute pharmacological action and instead suggests durable physiological remodeling. In particular, prolonged colonization and metabolic activity of probiotic strains may sustain local intestinal effects that continue to modulate systemic oxygen availability.

Current preventive strategies for altitude illness have recognized limitations: staged ascent does not guarantee everyone the same acclimatization, and acetazolamide, although effective, may carry contraindications and side effects that limit applicability in up to 30% of at-risk populations. The present study establishes proof of concept for a non-pharmacological, broadly tolerable adjunct that could be integrated into existing prophylactic protocols without replacing essential acclimatization processes. The absence of statistically significant reduction in AMS incidence, despite substantial improvements in SpO_2_, merits careful interpretation and should not overshadow the primary physiological findings. In fact, AMS represents a complex, multifactorial syndrome influenced by ascent rate, genetic predisposition, fluid balance, and individual physiological reserve.^14^^,^^15^^,^^43^ The universal administration of acetazolamide prophylaxis in the present study, while ethically necessary, introduced a powerful confounding factor that may have obscured treatment effects on AMS incidence. On the other hand, oxygen saturation represents a more objective, mechanistically proximate outcome that better captures the intervention’s core physiological effect, and the observed preservation of SpO_2_ above critical thresholds likely translates to reduced symptom burden even in the absence of categorical differences in AMS diagnosis. However, future studies in drug-free cohorts are needed to fully evaluate the role of the probiotic in preventing AMS.

The study suggests that Oxxyslab supplementation may offer a protective role against oxygen saturation drops in severe hypoxic conditions, even during intense physical exercise and in individuals of different ethnic origins, supporting a novel approach to the management of high-altitude hypoxia. Modulating the gut microbiota may serve as a clinically relevant adjunct to established strategies, while not substituting for acclimatization processes, which remain indispensable.

The inclusion of probiotics within preventive protocols for individuals exposed to high altitude appears feasible. Further clinical investigations involving broader cohorts and diverse environmental conditions will be essential to delineate more precisely the clinical relevance and translational potential of this strategy.

### Limitations of the study

All Italian participants received prophylactic acetazolamide to mitigate AMS symptoms.^13^^,^^14^ This medication could have influenced the incidence and severity of AMS, thereby acting as a potential confounding factor. Moreover, the inherent subjectivity of the Lake Louise score makes it susceptible to methodological biases; factors such as self-administration versus investigator-assisted evaluation, the frequency of testing, and the concomitant use of medication can significantly alter symptom perception and reporting.^44^

Although the sample size is relatively small, the study remains highly relevant given the particular conditions in which it was conducted. However, a direct comparison between lowlanders and Nepalese porters was not possible due to confounding factors such as diverse genetic backgrounds, different timelines of probiotic administration and duration of SpO_2_ recording, and differences in diet and drug use between the two groups.

Finally, as a clinical field trial, this study was not designed to directly measure mechanistic endpoints such as splanchnic blood flow, intestinal oxygen extraction, or systemic HIF-1α stabilization. The proposed mechanisms linking probiotic supplementation to improved systemic oxygenation remain hypothetical and warrant dedicated investigation in controlled experimental models.

## Resource availability

### Lead contact

Further information and requests for resources should be directed to and will be fulfilled by the lead contact, Vittore Verratti (vittore.verratti@unich.it).

### Materials availability

This study did not generate new unique reagents.

### Data and code availability

• Due to privacy and ethical restrictions regarding human subjects data, as stipulated by the Ethics Review Board of the Nepal Health Research Council (NHRC) and the local Ethics Committee, raw datasets cannot be made publicly available. However, de-identified clinical data reported in this paper will be shared by the lead contact upon reasonable request.
• All original code and scripted pipelines used for the statistical analyses are available from the lead contact upon request.
• Any additional information required to reanalyze the data reported in this paper is available from the lead contact upon request.

## Acknowledgments

We are grateful to Going Nepal Pvt. Ltd. and the OMKAAR Polyclinic Medical Diagnostic and Therapeutic Center in Kathmandu for their invaluable logistical assistance. We extend our sincere appreciation to all trekkers and porters who contributed to the operational success of this project. A special acknowledgment is reserved for the EV-K2-CNR Association, in particular to Agostino Da Polenza, for their continued support and commitment to high-altitude research.

## Author contributions

V.V. planned and conducted the experiments and literature review, harmonized the data, wrote the first draft of the paper, and had overall responsibility for the paper. M. Marazzato planned and conducted the experiments and literature review. P.P. conducted the literature review and interpreted the results. K.F.A.B. conducted the literature review and interpreted the results. G.V. conducted the experiments. S.M.-S. interpreted the results and revised the draft. M. Marzola conducted the experiments and preliminary analyses, interpreted the results, contributed to drafting the paper, and revised the draft.

## Declaration of interests

The authors declare no conflict of interest.

## STAR★Methods

### Key resources table

**Table:** 

REAGENT or RESOURCE	SOURCE	IDENTIFIER
**Software and algorithms**		

Python (v3.11)	Python Software Foundation	https://www.python.org; RRID: SCR_008394
statsmodels library	statsmodels developers	https://www.statsmodels.org; RRID: SCR_016074
seaborn library	seaborn developers	https://seaborn.pydata.org; RRID: SCR_018132
Canva	Canva	https://www.canva.com

### Experimental model and study participant details

All participants provided written informed consent to participate in the study. This research was approved by the Ethics Review Board of the Nepal Health Research Council (NHRC, ref. no. 1084). and received approval from the local Ethics Committee (Ethics Committee of Province di Chieti e Pescara, document no. 18, 29/07/2021). All study procedures conformed to the ethical standards set by the Declaration of Helsinki and its subsequent amendments.^45^

The study includes 21 Italian Caucasian adults (7 females and 14 males; mean age 36.9 ± 12.8, range 21–56; mean BMI 24.1 ± 3.1 kg/m^2^) and 8 Nepalese porters (8 males; mean age 31.3 ± 7.32, range 20–38). Italian Caucasian participants took part in the “*Pyramid 24 Exploration and Physiology*” expedition to Sagarmatha (Mount Everest), Nepal, which took place from October 21 to November 14. The expedition involved a trekking route of 121 km with a total altitude variation of 6,521 m (Figure 3). Nepalese porters joined the expedition exclusively during the trekking phase, from October 25 to November 7. The Nepalese cohort was recruited from the Khumbu Valley and comprised individuals of Rai and Tamang ethnicity, all of whom had substantial experience working at high altitudes.

**Figure:**
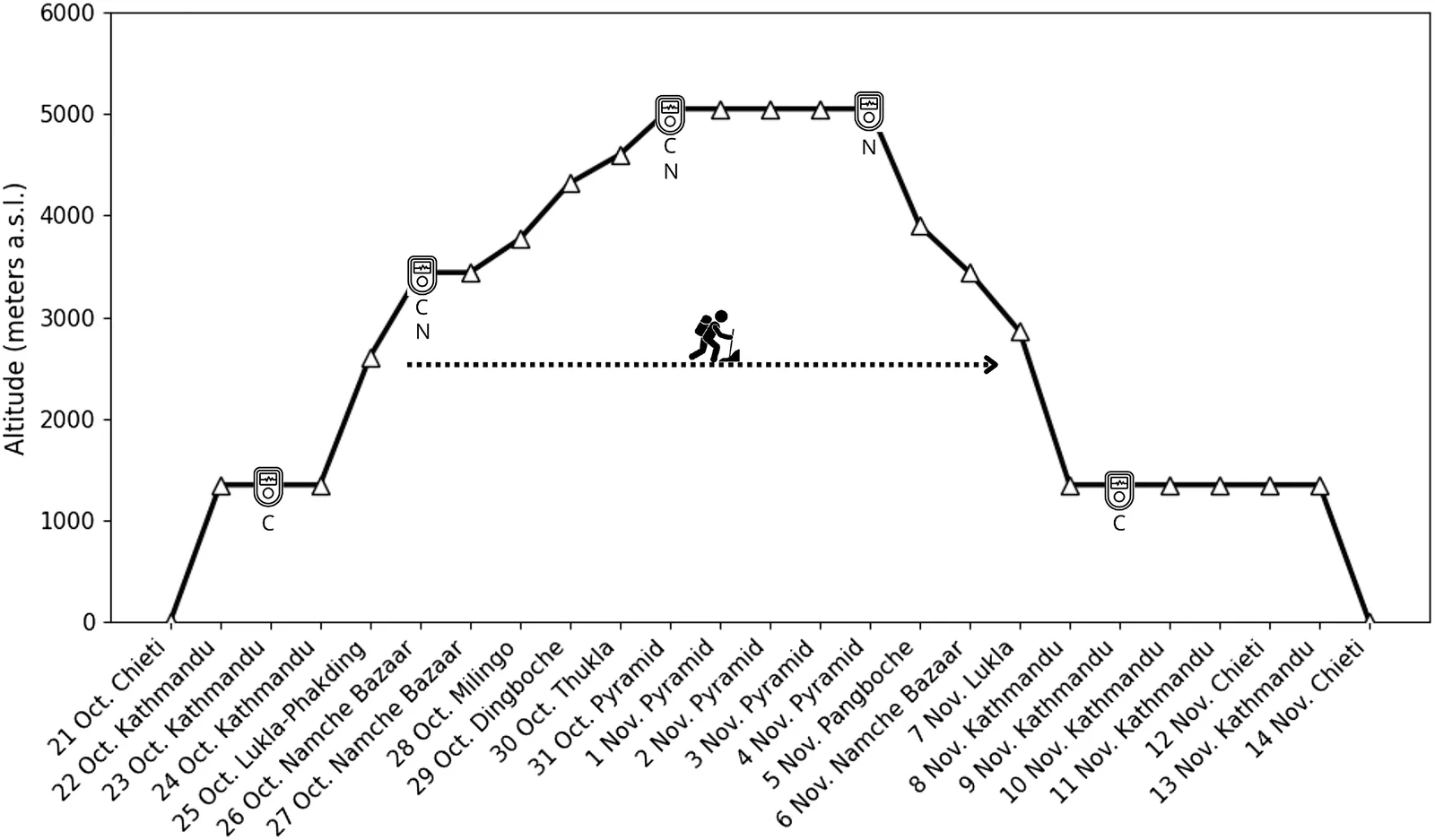
Altimetric scheme of the “Pyramid 24 Exploration and Physiology” expedition The trekking route to Sagarmatha (Mount Everest), Nepal, covering 121 km with a total altitude variation of 6,521 m. The icons denote the respective time points of assessment for nocturnal oxygen saturation: four points for the Italian Caucasians (C) and three points for the Nepalese porters (N). The scheme maps the specific altitudes and corresponding study phases (baseline, mid-altitude, peak altitude, and post-trek recovery) where physiological and clinical data were collected.

Given the limited sample size and the gender composition of the cohorts, the study was not sufficiently powered to perform stratified analyses to assess the independent influence of sex or gender on the study outcomes.

### Method details

Before the expedition, Italian participants were matched in pairs based on age and gender, and randomly assigned to either the placebo group (PG) or the treatment group (TG) through balanced randomization conducted by an external assistant using dedicated software (randomization.org). Each dose of probiotic or placebo was packaged in identical white sachets. Before departure, the same external assistant, who was not involved in data collection or analysis, prepared and labeled individual boxes for each participant according to their group allocation. This assistant was the only person aware of the group assignments. During the expedition, the boxes were distributed to participants without revealing their contents. Nepali porters were randomly assigned to PG or TG and received their daily sachets from a member of the expedition team. The research team remained blinded to group allocation until after data collection and the completion of preliminary analyses.

In detail: each sachet contained 6 g of powder mixture. The probiotic formula Oxxyslab contains about 8 × 10^11^ live bacteria per dose, comprising *Streptococcus thermophilus* CNCM I-5570, *Lactobacillus acidophilus* CNCM I-5567, *Lactobacillus plantarum* CNCM I-5569, *Lactobacillus paracasei* CNCM I-5568, *Lactobacillus helveticus* CNCM I-5573, *Lactobacillus brevis* CNCM I-5566, *Bifidobacterium animalis* subsp. *lactis* CNCM I-5571, *Bifidobacterium animalis* subsp. *lactis* CNCM I-5572. The placebo consisted of all components present in the probiotic formulation except for the bacteria, which were replaced with additional maltose.

Italian participants consumed either a probiotic or a placebo within 10 min following lunch and dinner each day of the study. The administration protocol began on the 1^st^ day of trekking and continued until the 2^nd^ day after reaching peak altitude, for a total duration of 8 days. Nepali porters received a double dose each morning prior to the start of trekking, beginning the day after the trek commenced and continuing until the third day post-peak altitude, totaling 8 days. To minimize dietary variability, Italian participants assumed the same meals throughout the study. Furthermore, no fermented foods were ingested. Each Italian participant (treated and control) took 125 mg of Diamox twice daily, morning and evening, during ascent, on the second day of the trek, until the second day of peak altitude reached. Nepalese porters did not assume any drugs.

To standardize measures across participants, SpO_2_ and HR were monitored by a pulse oximeter (Checkme O2, Viatom Technology, Shenzhen, GD) during the entire sleep period. Each participant was instructed to wear the device shortly before falling asleep and to remove it immediately upon waking. They were also instructed to maintain their habitual pre-sleep routines before data acquisition and to achieve a sleep duration of approximately 6–7 h. Italian trekkers were assessed at four time points: at baseline altitude in Kathmandu (I-T0), at mid-altitude in Namche (I-T1), at high altitude at the Pyramid Laboratory (I-T2), and upon return to baseline altitude (I-T3). Nepali trekkers were assessed at their habitual altitude of residence in Namche (N-T0), as well as on the first and last days spent at high altitude (N-T1 and N-T2). The experimental timeline is shown in Figure 3. Probiotic administration timing and measurement schedules between groups emerged for both physiological and logistical field reasons. Particularly, baseline living altitudes of the groups and specific expedition work duties for Nepalese workers, which require adaptations and preclude perfect synchronization between cohorts. To ensure data quality and minimize potential bias, each recording was carefully examined by an expert to identify and remove any anomalous values. According to AASM guidelines, 4 h was considered a minimum quality requirement in the Italian group. However, for the Nepalese porters, this continuous 4-h minimum could not be consistently achieved (resulting in the exclusion of N-T1) due to their specific expedition duties, which required significantly shorter sleep durations and earlier awakenings for camp management compared to the trekkers. In contrast, Nepalese porters didn’t require anomalous values cutting.

Three participants have been excluded from the SpO_2_ analysis because their basal recordings showed desaturation events compatible with pre-existing nocturnal apneas. This exclusion was implemented to avoid potential bias due to respiratory conditions that affect oxygen saturation.

Acute Mountain Sickness (AMS) was assessed using the 2018 Lake Louise Questionnaire (LLQ) at three key points during ascent: Namche (3450m), Dingboche (4320m), and the Pyramid International Laboratory/Observatory (5050m). According to the established recommendations, the assessments were conducted approximately 6 h after arrival at each designated altitude. AMS was considered present when the total score was ≥3, including at least one point attributed to headache.^44^

### Quantification and statistical analysis

All analyses were conducted using Python (v3.(11) with statsmodels and seaborn libraries.

We fit a series of linear mixed-effects models (LMMs) to estimate changes in SpO_2_ over time within the Italian cohort. The model was specified as SpO^2^_perc C(time) + treatment + C(time): treatment with random intercepts for each participant (subject) to account for within-subject correlation. We computed z-tests on fixed-effect coefficients and used heteroscedasticity-robust standard errors.

For the Italian cohort, we performed model-based post hoc contrasts on the fixed-effect estimates from the mixed-effects model. Specifically, we extracted the variance-covariance matrix of the estimated coefficients and computed pairwise contrasts between time-related parameters, separately for the main effects of time (i.e., differences between time levels at baseline) and for the time-by-treatment interaction terms (i.e., differences in treatment-related slopes across time points). Standard errors for each contrast were derived analytically using the delta method, and *p*-values were obtained using two-sided t-tests. To correct for multiple comparisons across all time point combinations, these *p*-values were adjusted using the False Discovery Rate (FDR) method (Benjamini-Hochberg procedure). All tests were adjusted for uncertainty in the fixed-effect estimates by incorporating the full covariance structure estimated from the model. This allowed direct model-based comparison of all time points, including non-adjacent levels, while maintaining the dependency structure of the repeated measures. For the Nepalese porters (Rai and Tamang), where only two time points were available, no post hoc contrasts were required, and therefore, multiple comparisons correction was not applicable. The same model structure was used to estimate main and interaction effects of time and treatment, with random intercepts by subject to account for clustering.

Finally, to assess the robustness of our findings and ensure that individual outlier participants did not drive significant results, Leave-One-Out (LOO) sensitivity analyses were conducted for both cohorts. This procedure involved systematically excluding one subject at a time and re-fitting the linear mixed-effects models on the remaining data to evaluate the stability of the treatment × time interaction coefficients.

LLQ positivity was analyzed by a generalized linear mixed-effects model using a logit link function. Wald tests on fixed-effect coefficients were performed to assess statistical significance.

Statistical significance was defined as a two-sided *p*-value <0.05. No corrections were applied for multiple testing in exploration post hoc contrasts. All analyses were pre-specified and reproducible using scripted pipelines.
