# Supplementary material for: Probiotic supplementation preserves oxygen saturation during severe hypobaric hypoxia in lowland and high-altitude populations
Source: iScience. 2026 Aug 27;29(9):117305. doi: 10.1016/j.isci.2026.117305 (PMC13546876; doi:10.1016/j.isci.2026.117305)
Supplement: Supplementary file 1 [file mmc1.pdf]

iScience, Volume *29*

Supplemental information

Probiotic supplementation preserves oxygen saturation during severe hypobaric hypoxia in lowland and high-altitude populations

Vittore Verratti, Massimiliano Marazzato, Pierpaolo Prosperi, Kiara Fiorella Abad Bruzzo, Gianpietro Verza, Simona Mrakic-Sposta, and Manuel Marzola

SUPPLEMENTARY INFORMATION

Supplementary Figure 1: *Sensitivity analysis for the Italian cohort. Leave-One-Out (LOO) plot showing the stability of the treatment × time interaction coefficient at peak altitude (I-T2) across all 21 iterations. Each dot represents the model estimate after excluding one subject.*

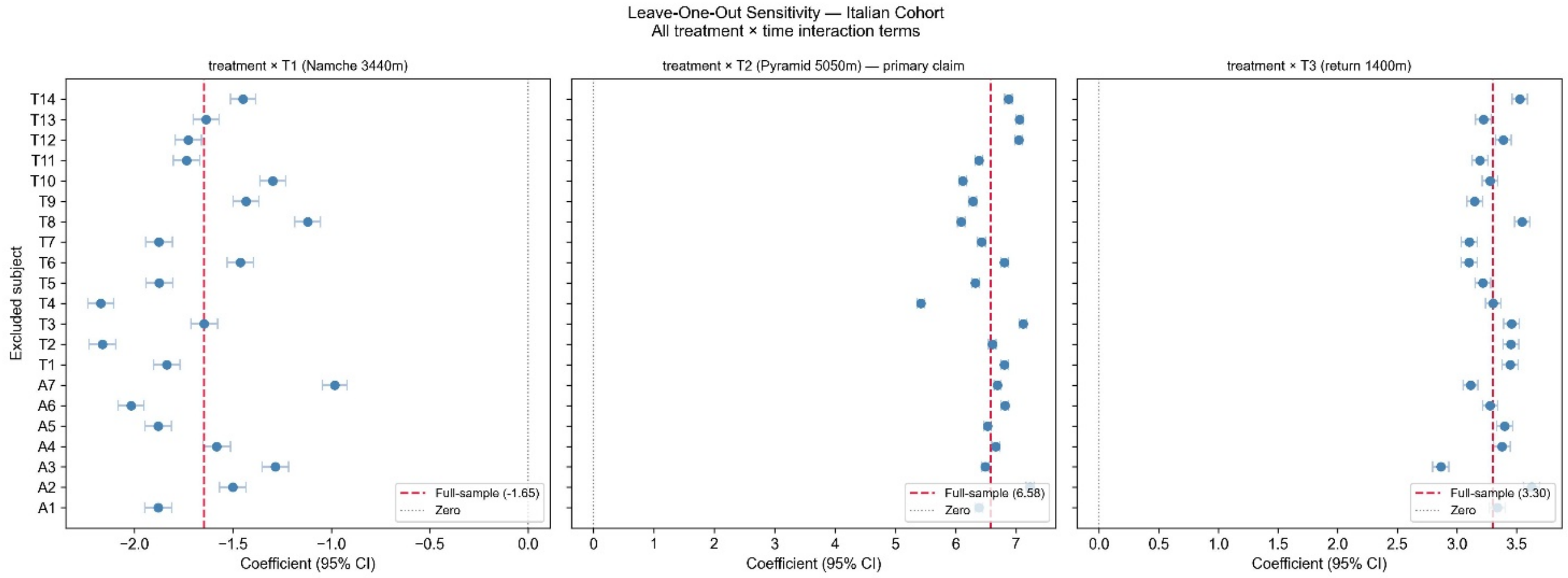

Supplementary Figure 2: *Sensitivity analysis for the Nepalese porter cohort. Leave-One-Out (LOO) plot showing the stability of the N-T2 interaction coefficient across all 8 iterations.*

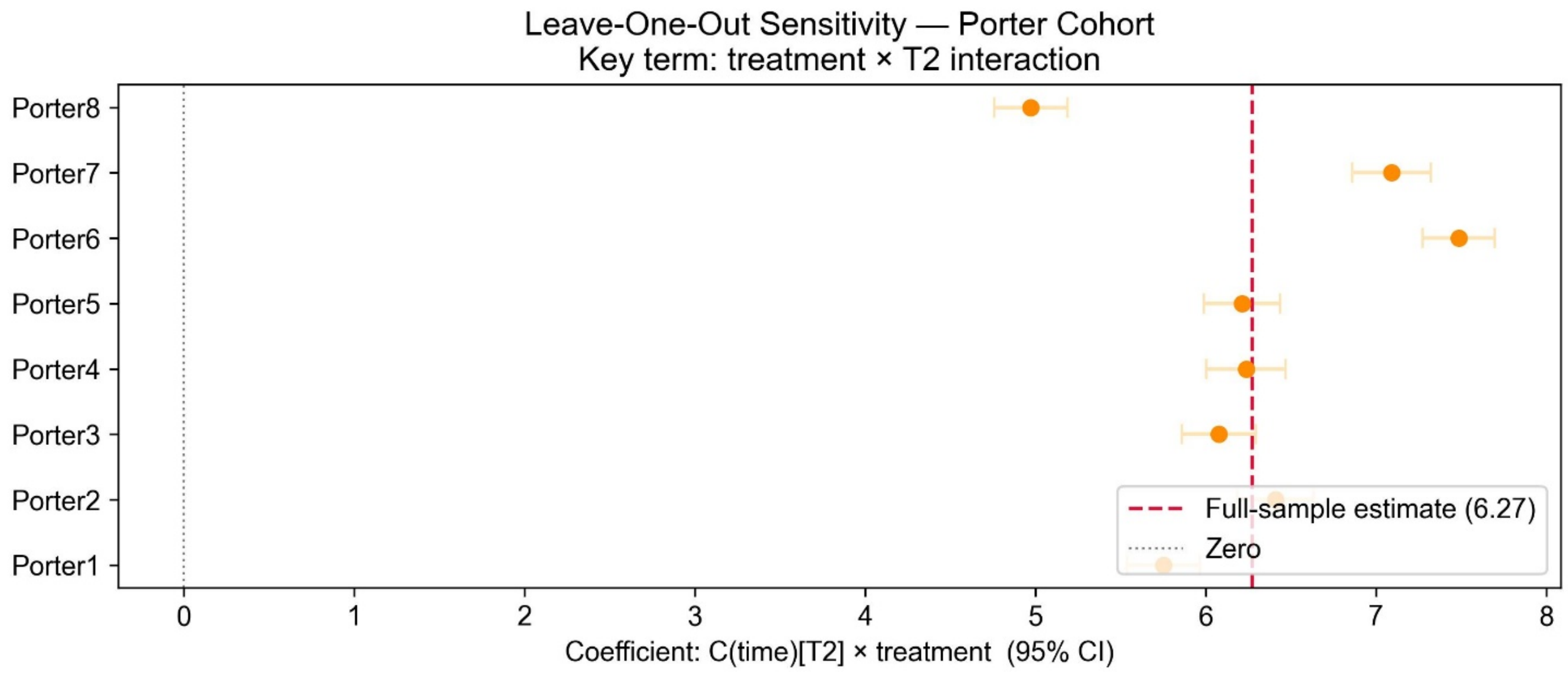

Supplementary Table 1. Linear Mixed-Effects Model for SpO_2 changes over time and treatment interactions (Italian Cohort)

**Parameter**	**Beta**	**Standard Error (SE)**	**Lower-95**	**Upper-95**	**p-value**	**Random Effect**
Intercept (Baseline, Placebo: I-T0)	95.044	0.744	93.585	96.503	< 0.001	
Time: Namche (I-T1)	-9.213	0.024	-9.260	-9.166	< 0.001	
Time: Peak Altitude (I-T2)	-20.311	0.023	-20.355	-20.266	< 0.001	
Time: Kathmandu Post (I-T3)	-0.110	0.024	-0.156	-0.063	< 0.001	
Treatment (Treated Group)	-1.002	1.028	-3.017	1.014	0.330	
Time (I-T1) × Treatment	-1.647	0.033	-1.712	-1.582	< 0.001	
Time (I-T2) × Treatment	6.578	0.031	6.516	6.639	< 0.001	
Time (I-T3) × Treatment	3.301	0.033	3.237	3.365	< 0.001	
Subject Intercept						2.353

Supplementary Table 2A. Linear Mixed-Effects Model for SpO_2 changes over time and treatment interactions (Nepalese Porters)

**Parameter Name**	**Beta**	**Standard Error (SE)**	**Lower-95**	**Upper-95**	**p-value**	**Random Effect**
Intercept (Baseline, Placebo: N-T1)	92.185	0.374	91.451	92.918	< 0.001	
Time: Peak Altitude (N-T2)	-7.682	0.074	-7.827	-7.537	< 0.001	
Treatment (Treated Group)	-2.063	0.529	-3.100	-1.026	< 0.001	
Time (N-T2) × Treatment	6.275	0.105	6.070	6.480	< 0.001	
Subject Intercept						0.741
